# Dipeptidyl peptidase like 6 promoter methylation is a potential prognostic biomarker for pancreatic ductal adenocarcinoma

**DOI:** 10.1042/BSR20200214

**Published:** 2020-07-30

**Authors:** Xin Zhao, Di Cao, Zhangyong Ren, Zhe Liu, Shaocheng Lv, Jiqiao Zhu, Lixin Li, Ren Lang, Qiang He

**Affiliations:** Department of Hepatobiliary Surgery, Beijing Chao-Yang Hospital Affiliated to Capital Medical University, Beijing, China

**Keywords:** methylation, pancreatic ductal adenocarcinoma, promoter, TCGA

## Abstract

**Background:** Hypermethylation of gene promoters plays an important role in tumorigenesis. The present study aimed to identify and validate promoter methylation-driven genes (PMDGs) for pancreatic ductal adenocarcinoma (PDAC). **Methods:** Based on GSE49149 and the PDAC cohort of The Cancer Genome Atlas (TCGA), differential analyses of promoter methylation, correlation analysis, and Cox regression analysis were performed to identify PMDGs. The promoter methylation level was assessed by bisulfite sequencing polymerase chain reaction (BSP) in paired tumor and normal tissues of 72 PDAC patients. Kaplan−Meier survival analyses were performed to evaluate the clinical value of PMDGs. **Results:** In GSE49149, the β-value of the dipeptidyl peptidase like 6 (*DPP6*) promoter was significantly higher in tumor compared with normal samples (0.50 vs. 0.24, *P*<0.001). In the PDAC cohort of TCGA, the methylation level of the *DPP6* promoter was negatively correlated with mRNA expression (r = −0.54, *P*<0.001). In a multivariate Cox regression analysis, hypermethylation of the *DPP6* promoter was an independent risk factor for PDAC (hazard ratio (HR) = 543.91, *P*=0.002). The results of BSP revealed that the number of methylated CG sites in the *DPP6* promoter was greater in tumor samples than in normal samples (7.43 vs. 2.78, *P*<0.001). The methylation level of the *DPP6* promoter was moderately effective at distinguishing tumor from normal samples (area under ROC curve (AUC) = 0.74, *P*<0.001). Hypermethylation of the *DPP6* promoter was associated with poor overall (HR = 3.61, *P*<0.001) and disease-free (HR = 2.01, *P*=0.016) survivals for PDAC patients. **Conclusion:** These results indicate that *DPP6* promoter methylation is a potential prognostic biomarker for PDAC.

## Background

Pancreatic ductal adenocarcinoma (PDAC) is a lethal malignant tumor with low excision rate, poor overall survival (OS), and high metastatic incidence [1]. Up until now, radical resection was the only possible curative treatment for PDAC. However, most pancreatic cancer (PC) patients were diagnosed in the progressive stage and missed the opportunity for curative surgery. Therefore, identification of tumor-specific diagnostic and prognostic biomarkers is beneficial for the early treatment of PDAC. Recent studies indicated that during the pancreatic carcinogenesis, molecular epigenetic alterations are driving factors that have potential applications in early diagnosis and survival prediction [2].

DNA methyltransferases 1/3A/3B promote the addition of a methyl moiety of S-adenosylmethionine to the 5′ position of a cytosine residue in CpG dinucleotides. DNA regions that are rich in CpG sites are called CpG islands. CpG islands can be found in 40–60% of gene promoter regions, and play an important role in regulating gene expression. Genomic hypomethylation and promoter hypermethylation are typical epigenetic features during cancerization or aggression. In human malignancy, hypermethylation at promoter-associated CpG islands, which generally inhibits gene expression, has been proven to be a hallmark epigenetic alteration [3–6].

With regard to PDAC, aberrant methylation of gene promoters was found to be involved in oncogenesis and progression. For example, promoter hypermethylation of *APC* was detected in the pancreatic juice of PDAC patients [7]. In the pancreatic microenvironment, the promoter of *SOCS1*, which encodes a member of the suppressor of cytokine signaling family, is frequently methylated in cancer-associated fibroblasts [8]. Similarly, *SOCS3* is also hypermethylated and leads to PC growth and metastasis by activating the IL-6 signal transducer and the *STAT3* signaling pathway [9]. Finally, promoter methylation-based biomarkers such as *EFEMP1* can predict the malignant formation of pancreatic precancerous lesions [10].

**Figure 1:**
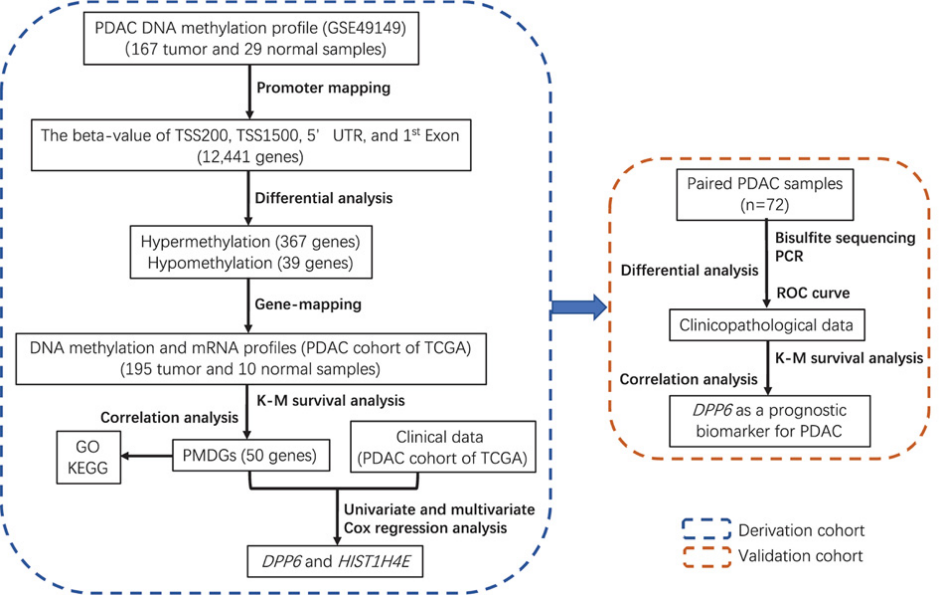
The flowchart of the present study Abbreviations: GSE49149, genome-wide DNA methylation patterns in PDAC (accession number of GEO, 49149); GO, Gene Ontology; KEGG, Kyoto Encyclopedia of Genes and Genomes; K–M survival analysis, Kaplan–Meier survival analysis; PCR, polymerase chain reaction; ROC curve, receiver operating characteristic curve; TCGA, The Cancer Genome Atlas; TSS, transcription start site; UTR, untranslated region.

Some tools and algorithms have been developed to identify methylation-driven genes for cancers [11,12]; however, the relationship between gene promoter methylation level, mRNA expression, and clinical phenotype was poorly explored, especially for PDAC. The aim of the present study was to identify promoter methylation-driven genes (PMDGs), which were aberrantly methylated in the promoter region, negatively correlated with mRNA levels, and associated with OS for PDAC. Potential PMDGs were first screened with public databases as a derivation cohort and then validated with our own datasets (Figure 1).

## Materials and methods

### DNA methylation datasets of the derivation cohort

In the derivation cohort, the DNA methylation profile (accession number: GSE49149) was downloaded from the Gene Expression Omnibus (GEO) database (https://www.ncbi.nlm.nih.gov/geo/) [13,14]. The level-3 DNA methylation dataset of the PDAC cohort was downloaded from the Broad TCGA (The Cancer Genome Atlas) GDAC (http://gdac.broadinstitute.org). Both sets of methylation data were screened with the HumanMethylation450 (HM450K) Illumina SNPBeadChip and scanned with iScan. Between the two DNA methylation databases, the β-value, which is the ratio of the methylated probe intensity and the overall intensity, was applied to describe the methylation degree. The β-value ranged from 0 to 1, and a higher β-value represented higher methylation.

### mRNA sequencing and clinical data of the PDAC cohort of TCGA

Publicly available mRNA level-3 sequencing of the PDAC cohort of TCGA was obtained from GDAC, and a normalized RSEM count value indicated gene expression. We processed the RSEM count value with log_2_ and removed 10% minimal expression genes in all samples. We extracted clinicopathological parameters including age, postoperative chemotherapy, neoplasm recurrence, resection status, tumor dimension, and lymph node metastasis from the clinical data of the PDAC cohort of TCGA. Follow-up times and survival conditions were also acquired to perform the Kaplan–Meier (K–M) survival analysis and Cox regression analysis.

### Identification of PMDGs

In GSE49149, we first screened for the probe ID in four promoter regions, including transcription start site (TSS) 200 (TSS200), TSS1500, 5′ untranslated regions (5′UTR), and the first exon (1st exon). Since multiple CpG islands were detected in one promoter region of most genes, we averaged the β-values in this region to describe the methylation status. We then chose the β-value of TSS200 to represent the promoter methylation status for one gene. If there were no CpG islands in the TSS200 for a gene, we chose the β-values of TSS1500, 5′UTR, or 1st exon to represent the methylation status for this gene. Finally, we converted the β-value into log_2_(β-value) and employed the Limma package to identify differentially methylated genes under the threshold (|log_2_ fold change (log_2_ FC)| >1 and *P*<0.05) [15].

In the PDAC cohort of TCGA, the Pearson correlation was implemented to evaluate the relevance between the promoter methylation level and mRNA expression value. A negative correlation was considered significant if the *P*-value was less than 0.05. To screen out survival-associated genes, we divided the patients into high-expressed and low-expressed groups according to the median value of mRNA expression and performed a Kaplan–Meier survival analysis (K–M survival analysis). To eliminate the impact of surgical complications on survival, we excluded cases where the death occurred within 90 days postoperatively. In the PDAC cohort, 163 patients were enrolled in the survival analysis.

To understand the function of PMDGs, we applied the clusterProfiler package to analyze the Gene Ontology (GO) annotation and Kyoto Encyclopedia of Genes and Genomes (KEGG) pathway enrichment [16].

To identify independent factors for OS, we performed a univariate and multivariate Cox regression analyses combining the clinical parameters with the methylation values of the PMDGs.

### Patients in the validation cohort

Between September 2016 and December 2018, 72 patients with PDAC who underwent pancreatoduodenectomy and diagnosed by histological evidence postoperatively, were enrolled in the validation cohort. The clinicopathological characteristics of these patients were collected, and the survival status was followed-up till September 2019. The patients were followed up for an average of 14 months (2–34 months). The present study was permitted by the research ethics committee of Chao-Yang Hospital, and all patients signed the informed consent (No. 2017-S-241).

### Bisulfite sequencing polymerase chain reaction

In the validation cohort, 72 pairs of tumor and adjacent normal pancreatic tissues were collected from PDAC patients, and the CG sites of the promoter regions of four potential PMDGs were examined using bisulfite sequencing polymerase chain reaction (BSP). First, the upstream 3-kb DNA sequence of the gene promoter region was extracted from the NCBI dataset (Supplementary Figure S1). We predicted the CpG island with MethPrimer and selected the CpG island closest to the location of the probe in methylation microarrays (Supplementary Figure S2) to perform the subsequent cloning sequencing. The primer was designed with Primer5 software V5.6 (Table 1) according to the sequence of the CpG island. After that, the Universal DNA Purification Kit (DP214; Tiangen, Beijing, China) was used to extract and purify DNA from tissue samples following the manufacturer’s manual. The EZ DNA Methylation-Direct Kit (D5020; Zymo Research, CA, U.S.A.) and pEASY®-T1 Cloning Kit (CT101-01; TransGen Biotech, Beijing, China) were used to perform the DNA bisulfite conversion and polymerase chain reaction (PCR) cloning sequencing. We used the BiQ Analyzer to analyze the original sequencing data and performed a comparison of methylated CG sites (Supplementary Figure S3).

**Table 1:** The primer sequence of four PMDGs

Gene	Primer sequence (5′–3′)	Product size (bp)
*DPP6*-F	TTG(C/T)GTT(C/T)GTTTAATTTTGATGTAG	512
*DPP6*-R	AAAAATCTTCCAAATCTTCAATT	
*ZFP28*-F	TATTTTGGAGGATGGGAGGTT	389
*ZFP28*-R	TAACCAAC(G/A)CTAAACCTAAATATAAC	
*MTMR7*-F	GGGAAAAGTGT(C/T)GTTTGTAATAGTG	411
*MTMR7*-R	CTAAAATTACC(G/A)AAAC(G/A)AAAACTACT	
*HIST1H4E*-F	ATTTTATTTAGTTGTTAAAATATGTT	373
*HIST1H4E*-R	AACTTAATAATACCCTAAATATTATCT	

Abbreviations: *DPP6*, dipeptidyl peptidase like 6; *HIST1H4E*, H4 clustered histone 5; *MTMR7*, Myotubularin related protein 7; *ZFP28, ZFP28* zinc finger protein.

### Validation of the PMDGs as potential biomarkers for PDAC

A differential analysis of the promoter methylation levels of PMDGs was performed between pancreatic tumor and adjacent normal tissue samples. A receiver operating characteristic (ROC) curve was then constructed based on the numbers of methylated CG sites of the promoter. The area under the ROC curve (AUC) was calculated to evaluate the efficiency of methylation levels in distinguishing tumor from normal samples. According to the median value of the numbers of the methylated CG sites, the PDAC patients were divided into high-level and low-level groups. A K–M survival analysis was performed to evaluate the OS between the two groups. The clinicopathological parameters were also compared between the two groups.

### Statistical analysis

R software version 3.6.0 was used to generally integrate and analyze data. The Limma package was employed to identify the differentially methylated promoters. The Pearson correlation analysis between promoter methylation level and mRNA expression, and a K–M survival analysis were used to identify the PMDGs. The R packages of pheatmap and ggplot were used to complete the visualization of the results. Univariate and multivariate Cox regression analyses were performed to highlight the potential prognostic biomarkers for PDAC. A paired-sample *t*-test and two-sample variance were used to compare continuous variables. The Wilcoxon signed ranks were utilized to analyze the correlation between gene expression level and clinicopathological parameters. *P*<0.05 was considered statistically significant.

## Results

### Identification of 50 PMDGs

**Figure 2:**
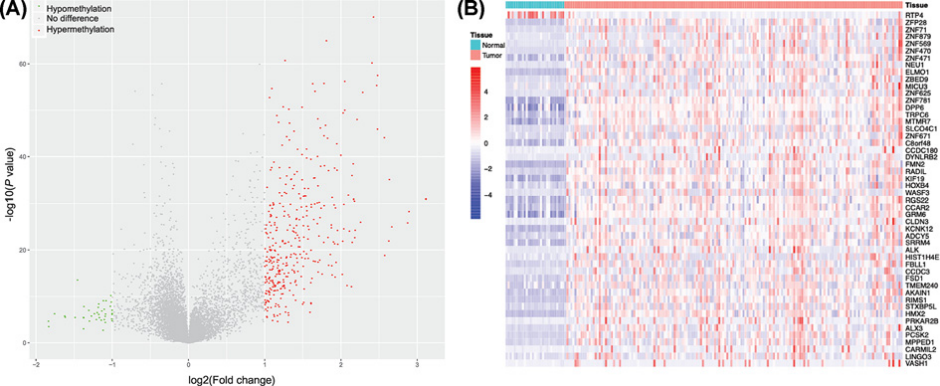
Identification of differentially methylated genes across the whole gene promoters (**A**) In GSE49149, a total of 367 hypermethylated genes (red dots) and 39 hypomethylated genes (green dots) were identified (|log_2_ FC| > 1 and *P*<0.05). (**B**) Heatmap of the 50 PMDGs. The mRNA values of PMDGs were negatively correlated with the promoter methylation levels (*P*<0.05), and the mRNA levels of PMDGs were associated with the OS of the PC cohort of TCGA (*P*<0.05). The red and blue bars on the top represent tumor and normal samples, respectively. Colors changing from mazarine (low) to crimson (high) represent the homogenized β-value of the gene promoter in GSE49149.

There were 167 malignant and 29 normal pancreatic samples in the GSE49149 dataset. In total, 406496 CpG sites were detected. The promoter regions included 27910, 34558, 16583, and 6418 CpG sites in TSS200, TSS1500, 5′UTR, and the 1st exon, respectively. After equalization, 9389 genes with CpG islands in TSS200, 11339 genes with CpG islands in TSS1500, 3281 genes with CpG islands in 5′UTR, and 3817 genes with CpG islands in 1st exon were confirmed. After integration, we obtained 12441 genes with average β-values in the promoter regions. We performed a differential methylation analysis and observed a total of 406 differentially expressed genes (|log_2_ FC| > 1 and *P*<0.05). Of these, 367 were hypermethylated, and 39 were hypomethylated in tumor tissues compared with normal tissues (Figure 2A).

To study the correlation between gene expression and promoter methylation, we downloaded the PDAC methylation data of TCGA, which included 185 malignant and ten normal samples. The probe IDs of TSS200, TSS1500, 5′UTR, and 1st exon for the 406 genes were mapped to the PDAC dataset of TCGA. After integrating the β-values of the four TSS regions into one, we obtained the methylation patterns for the 406 genes. We then screened out the mRNA expression count values of these genes from the PDAC mRNA sequencing data. Next, a Pearson correlation between the methylation value and mRNA expression of each gene was conducted, and 287 candidate genes were selected in which the correlation coefficient was significantly negative (*P*<0.05). Among these genes, 49 hypermethylated genes and one hypomethylated gene were significantly associated with OS and were identified as PMDGs (Figure 2B and Supplementary Table S1).

**Figure 3:**
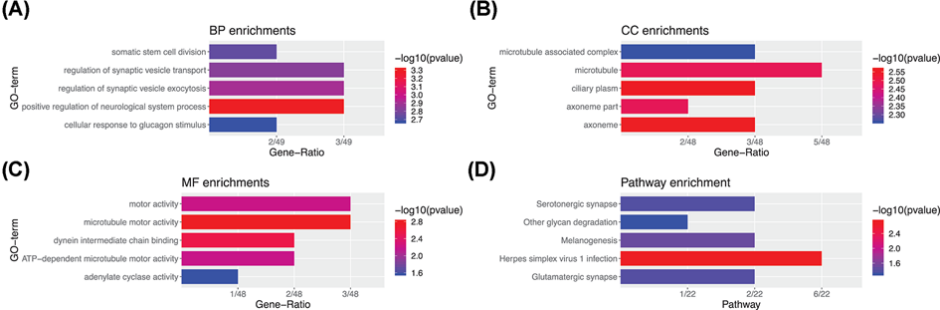
Functional enrichment analyses for the PMDGs Top five enriched GOs for the 50 PMDGs. (**A**) BP. (**B**) CC. (**C**) MF. (**D**) Top five signaling pathways enriched in KEGG for PMDGs.

GO analysis was employed to understand the functions of the 50 PMDGs. The results indicated that the top enriched term in biological process (BP) was ‘positive regulation of neurological process.’ The greatest gene number in cellular component (CC) was ‘microtubule.’ The most enriched term in molecular function (MF) was ‘microtubule motor activity’ (Figure 3A–C). Based on the KEGG pathway enrichment analysis, there were six genes participating in the pathway of herpes simplex virus-1 infection (*P*<0.01) (Figure 3D).

### Identification of potential prognostic biomarkers among PMDGs

**Figure 4:**
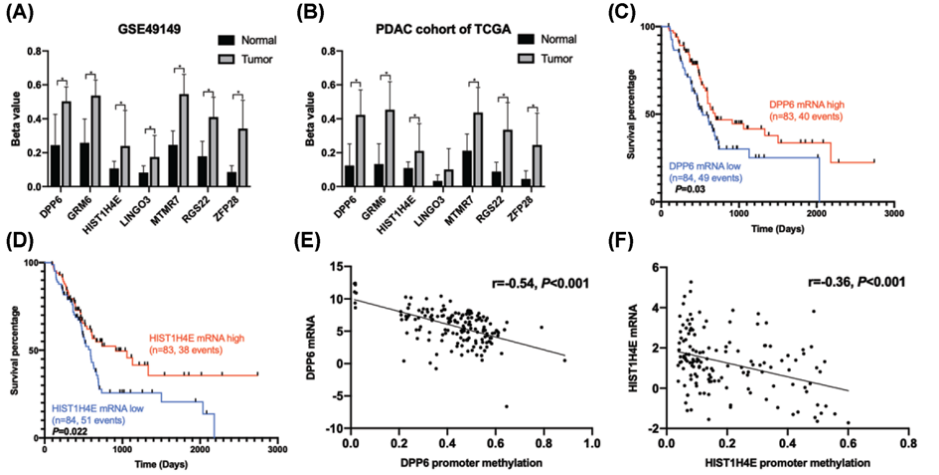
Identification of *DPP6* and *HIST1H4E* as prognostic biomarkers for PDAC (**A,B**) In the GSE49149 dataset and the PDAC cohort of TCGA, the promoter regions of seven PMDGs, including *DPP6, MTMR7, LINGO3, HIST1H4E, GRM6, ZFP28*, and *RGS22* were significantly hypermethylated in tumor compared with normal tissues (*P<0.05). (**C,D**) High mRNA levels of *DPP6* and *HIST1H4E* were associated with better OS of the PDAC cohort of TCGA. (**E,F**) The promoter methylation levels of *DPP6* and *HIST1H4E* were negatively correlated with mRNA expression values.

To determine the possible prognostic biomarkers in PMDGs, we extracted 14 clinicopathological variables from the PDAC cohort of TCGA, including age, therapy methods, and tumor histopathological characteristics. A univariate Cox regression analysis was performed based on methylation levels of the 50 PMDGs and the clinical parameters. After that, nine clinical characteristics, including age, postoperative chemotherapy, neoplasm recurrence, histological grade, use of molecular targeting drugs, radiotherapy, resection status, lymph node metastasis, and tumor grade showed statistical differences (Table 2). The promoter methylation level of seven PMDGs including dipeptidyl peptidase like 6 (*DPP6*), myotubularin related protein 7 (*MTMR7*), leucine rich repeat and Ig domain containing 3 (*LINGO3*), H4 clustered histone 5 (*HIST1H4E*), glutamate metabotropic receptor 6 (*GRM6*), *ZFP28* zinc finger protein (*ZFP28*), and regulator of G protein signaling 22 (*RGS22*) were significantly associated with OS (Table 2). In both the GSE49149 and PDAC cohort of TCGA, these seven genes showed similar methylation patterns in the promoter region: hypermethylation in tumor samples and hypomethylation in normal samples (Figure 4A,B).

**Table 2:** Univariate and multivariate Cox regression analyses according to clinicopathological variables and seven PMDGs in the PDAC cohort of TCGA

Clinical parameters	Univariate Cox regression analysis		Multivariate Cox regression analysis	
	HR (95% CI)	*P*	HR (95% CI)	*P*
Age	1.03 (1.00–1.05)	0.006	1.01 (0.98–1.04)	0.563
Resection status (R0, R1)	1.79 (1.17–2.74)	0.007	1.28 (0.80–2.00)	0.304
T stage (T1/T2, T3/T4)	2.01 (1.07–3.82)	0.031	1.32 (0.64–2.70)	0.455
Lymph node invasion (no, yes)	2.00 (1.19–3.37)	0.009	2.04 (1.11–3.80)	0.023
Histologic grade (Gx, G1, G2, G3, G4)	1.46 (1.08–1.98)	0.014	1.41 (0.99–2.00)	0.059
Neoplasm recurrence (no, NA, yes)	1.26 (1.01–1.58)	0.042	1.28 (0.99–1.70)	0.057
Molecular targeting drugs using (no, yes)	0.58 (0.38–0.88)	0.011	0.76 (0.42–1.40)	0.351
Radiotherapy (no, NA, yes)	0.72 (0.54–0.96)	0.024	0.70 (0.51–9.97)	0.034
Postoperative chemotherapy (no, yes)	0.58 (0.37–0.89)	0.013	0.38 (0.20–0.71)	0.003
*HIST1H4E*	14.11 (4.47–44.55)	0.000	5.92 (1.19–29.00)	0.030
*DPP6*	6.77 (1.70–27.00)	0.007	543.91 (9.50–31000.00)	0.002
*MTMR7*	5.66 (1.61–19.95)	0.007	4.00 (0.16–99.00)	0.397
*LINGO3*	9.17 (1.83–45.89)	0.007	7.01 (0.79–62.00)	0.08
*GRM6*	4.07 (1.15–14.49)	0.030	0.04 (0.00–1.40)	0.073
*ZFP28*	3.09 (1.06–9.02)	0.039	1.04 (0.20–5.30)	0.967
*RGS22*	3.48 (1.02–11.94)	0.047	0.05 (0.00–1.10)	0.054

Abbreviations: CI, confidence interval; HR, hazard ratio.

T stage is based on the 7th edition of the American Joint Committee on Cancer staging system.

We integrated the above-mentioned seven genes and nine clinical factors into a multivariate Cox regression analysis, and found that chemotherapy and radiation were significant protective factors, while lymph node metastasis and promoter hypermethylation of *DPP6* and *HIST1H4E* were significant risk factors for PDAC patients (Table 2). As previously mentioned, the K–M survival analysis showed that high mRNA levels of *DPP6* (hazard ratio (HR) = 0.63, 95% confidence interval (CI) = 0.42–0.96, *P*=0.03) and *HIST1H4E* (HR = 0.62, 95% CI = 0.41–0.93, *P*=0.02) were both associated with better OS (Figure 4C,D). Moreover, promoter methylation levels of *DPP6* and *HIST1H4E* were both negatively correlated with mRNA expression (Figure 4E,F).

### Validation of *DPP6* as a potential prognostic biomarker for PDAC

**Figure 5:**
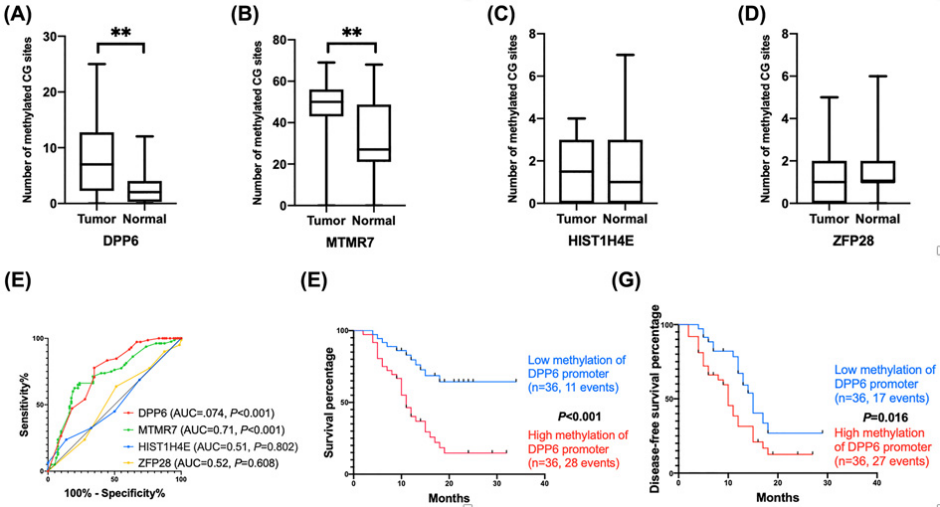
Validation of *DPP6* as a prognostic biomarker for PDAC (**A**–**D**) The number of methylated CG sites of the *DPP6* and *MTMR7* promoters was significantly greater in tumor than in adjacent normal samples. However, no difference was observed for *HIST1H4E* and *ZFP28*. (**E**) The number of methylated CG sites of the *DPP6* promoter could distinguish the tumor from normal samples with moderate efficiency (AUC = 0.74 and *P*<0.001). (**F,G**) Hypermethylation of the *DPP6* promoter was associated with poor overall and disease-free survivals for PDAC patients. **P<0.01.

BSP was performed focusing on the promoter regions of four potential PMDGs (*DPP6, HIST1H4E, MTMR7*, and *ZFP28*) in 72 paired tumor and adjacent normal samples of PDAC patients. The results confirmed that the numbers of methylated CG sites in *DPP6* and *MTMR7* in tumor tissues were significantly higher than in normal tissues (7.43 ± 6.12 vs. 2.78 ± 2.96, *P*<0.001; 45.95 ± 16.68 vs. 31.36 ± 16.90, *P*<0.001; Figure 5A,B). However, the numbers of methylated CG sites in *HIST1H4E* and *ZFP28* were not different between tumor and normal tissues (1.69 ± 1.43 vs. 1.74 ± 1.70, *P*=0.587 and 1.69 ± 1.37 vs. 1.47 ± 1.35, *P*=0.73; Figure 5C,D).

A ROC curve was constructed based on the number of methylated CG sites in *DPP6*. The results showed that the methylated status of the *DPP6* promoter had a moderate ability to distinguish tumor from normal tissues (AUC = 0.74, *P*<0.001; Figure 5E). The cut point was 4.5 methylated CG sites in the *DPP6* promoter, with a specificity of 65% and a sensitivity of 78%. Based on the median number of methylated CG sites of the *DPP6* promoter, we divided the patients into high- (*n*=36) and low-level (*n*=36) groups. The K–M survival analysis showed that the OS was significantly better in the low-level group than in high-level group (HR = 3.61, 95% CI = 1.91–6.84, *P*<0.001; Figure 5F). Similarly, the disease-free survival of the low methylation group was significantly better than in the high-level group (HR = 2.01, 95% CI = 1.11–3.64, *P*=0.016; Figure 5G).

### Association of the methylation level of the *DPP6* promoter with clinicopathological parameters of PDAC

Clinicopathological characteristics were compared between the above-mentioned high- and low-level groups (Table 3). Tumor size in the high-level group was significantly larger than the low-level group (4.34 vs. 3.34 cm, *P*=0.021). However, the ratio of R0 resection in the high-level group was lower than in the low-level group (94 vs. 64%, *P*=0.003). On the contrary, more patients in high-level group suffered from PDAC in the III or IV TNM stage compared with low-level group (92 vs 3%, *P*<0.001). Compared with the low-level group, the ratio of vascular invasion was higher in the high-level group, but not to a statistically significant level (53 vs. 33%, *P*=0.153).

**Table 3:** Kaplan–Meier survival analyses for PDAC patients stratified with clinicopathological parameters, and the association between the methylation level of the *DPP6* promoter and clinicopathological features

Clinical parameters	OS (30 months)			Methylation of *DPP6* promoter		
	*n*	HR (95% CI)	*P*	Low level (*n*=36)	High level (*n*=36)	*P*
Age (years)			0.599	63^1^	61^1^	0.522
≤62	36	1				
>62	36	1.18 (0.63–2.21)				
Tumor size (cm)			0.218	3.34^1^	4.34	0.021
<4	40	1				
≥4	32	1.47 (0.78–2.76)				
Resection			0.240			0.003
R0	57	1		34	23	
R1 and R2	15	1.71 (0.79–3.72)		2	13	
Invasion of reginal lymph node			0.234			<0.001
Negative	21	1		20	1	
Postitive	51	1.57 (0.79–3.15)		16	35	
Vascular invasion			0.243			0.153
Negative	41	1		24	17	
Postitive	31	1.44 (0.75–2.75)		12	19	
TNM stage			0.022			<0.001
II	38	1		35	3	
III and IV	34	2.37 (1.26–4.47)		1	33	
T stage			0.196			0.020
T1 and T2	50	1		30	20	
T3 and T4	22	1.51 (0.76–3.01)		6	16	
N stage			0.001			<0.001
N0 and N1	44	1		20	1	
N2	28	2.63 (1.35–5.12)		16	35	
M stage			0.575			0.011
Negative	65	1		36	29	
Postitive	7	1.30 (0.46–3.66)		0	7	

1 Mean value of continuous parameters.

TNM stage was based on the 8th edition of the American Joint Committee on Cancer staging system.

## Discussion

Hypermethylation in gene promoters is a general epigenetic modification in cancer formation, especially for inhibition of tumor-suppressive genes such as *PCDH10* [17], *DKK1* [18], and *KLF4* [19]. For example, *PCDH10* is down-regulated in PC cells and overexpression of *PCDH10* inhibits proliferation and migration of cells. Promoter methylation of *PCDH10* was observed in cancer cells, and the expression of *PCDH10* could be restored by 5-aza-2′-deoxycytidine [17]. For certain genes such as *CDO1*, promoter methylation could be used as a diagnostic biomarker, and methylation of the *ZNF671* promoter could predict survival [3,20].

In the present work, we focused on the methylation status of whole coding genes and found 50 PMDGs. Some of PMDGs were confirmed as tumor-suppressive genes, such as *VASH1* [21], *ALX3* [22], and *LDN3* [23]. Among the PMDGs, promoter hypermethylation of seven genes, *HIST1H4E, DPP6, MTMR7, LINGO3, GRM6, ZFP28*, and *RGS22*, were all correlated with poor survival of PDAC patients using a univariate Cox regression analysis. Most of the seven genes were associated with tumor suppression. *RGS22* was revealed to exhibit tumor suppressive function in hepatocellular carcinoma [24]. In a pancreatic cell line, overexpression of *RGS22* reduced cellular migration by coupling to GNA12/13, which led to inhibition of stress fiber formation [25]. Hypermethylation of *GRM6* was detected in renal carcinoma [26], and *LINGO3* was reported as one of the hub genes of metastatic melanoma [27]. In colorectal cancer, down-regulation of *MTMR7* was associated with a malignant phenotype by reducing the level of phosphoinositide and the activity of insulin-mediated AKT-ERK1/2 signaling [28].

The results of the multivariate Cox regression analysis showed that high levels of promoter methylation of *HIST1H4E* and *DPP6* were independent risk factors for PDAC. Therefore, we validated the methylation status of the two genes using BSP. The results confirmed that methylation levels of the *DPP6* promoter was significantly increased in tumor tissues compared with normal tissues. The number of methylated CG sites could be a potential biomarker to distinguish tumor from normal samples with moderate efficiency. The results of the K–M survival analysis verified that hypermethylation of the *DPP6* promoter was associated with poor OS and disease-free survival. Moreover, several malignant phenotypes, such as tumor size, lymph node invasion, and TNM stage, were related to hypermethylation of the *DPP6* promoter.

*DPP6* encodes a single-pass type II membrane protein, which binds to specific voltage-gated potassium channels and regulates dendritic excitability. GO annotations of *DPP6* include serine-type peptidase activity and dipeptidyl-peptidase activity. Somatic mutations of *DPP6* were discovered in PDAC [29], which suggests that its loss of function was associated with invasion of PC cells [30]. However, the function of *DPP6* in regulating tumor progression is unknown. Further studies are needed to define the potential molecular mechanism of this novel biomarker.

In renal cell carcinoma, the promoter hypermethylation of *DPP6* frequently occurred in tumor cells and was associated with poor survival serving as an independent predictor for distant metastasis [31]. Similarly, the promoter of *DPP6* was also found to be hypermethylated in a TCGA esophagus adenocarcinoma cohort [32]. To our knowledge, our study is the first to observe and validate that, the promoter of *DPP6* was significantly hypermethylated in PDAC, and that the number of methylated CG sites could be a prognostic biomarker for PC. Further molecular biological experiments are needed to reveal the function of *DPP6* in PDAC.

Furthermore, in the derivation cohort, the results of a multivariate Cox regression analysis revealed that postoperative chemotherapy and radiation were significant protective factors; however, lymph node metastasis was an independent risk factor for PDAC patients. This is accordance with the results of recent clinical cohort studies [33–35].

## Conclusions

We studied the promoter methylation status across the whole genome through a series of bioinformatics analyses and identified that hypermethylation of the *DPP6* promoter was an independent risk factor for PDAC. Using BSP and clinicopathological data of our own, we validated that as a PMDG, *DPP6* could be a potential prognostic biomarker for PDAC, which may provide a new therapeutic target for PC.

## Supplementary Material

Supplementary Figures S1-S3 and Supplementary Table S1

## Abbreviations

1st exon: first exon
AUC: area under ROC curve
BSP: bisulfite sequencing polymerase chain reaction
CI: confidence interval
*DPP6*: dipeptidyl peptidase like 6
FC: fold change
GO: Gene Ontology
*GRM6*: glutamate metabotropic receptor 6
*HIST1H4E*: H4 clustered histone 5
HR: hazard ratio
KEGG: Kyoto Encyclopedia of Genes and Genomes
K–M survival analysis: Kaplan–Meier survival analysis
*LINGO3*: leucine rich repeat and Ig domain containing 3
*MTMR7*: Myotubularin related protein 7
OS: overall survival
PC: pancreatic cancer
PDAC: pancreatic ductal adenocarcinoma
PMDG: promoter methylation-driven gene
*RGS22*: regulator of G protein signaling 22
ROC curve: receiver operating characteristic curve
TCGA: The Cancer Genome Atlas
TSS: transcription start site
UTR: untranslated region
*ZFP28*: *ZFP28* zinc finger protein
