# Supplementary material for: Dipeptidyl peptidase like 6 promoter methylation is a potential prognostic biomarker for pancreatic ductal adenocarcinoma
Source: Biosci Rep. 2020 Jul 30;40(7):BSR20200214. doi: 10.1042/BSR20200214 (PMC7396423; doi:10.1042/BSR20200214)
Supplement: Supplementary Figures S1-S3 and Supplementary Table S1 [file BSR-2020-0214_supp.pdf]

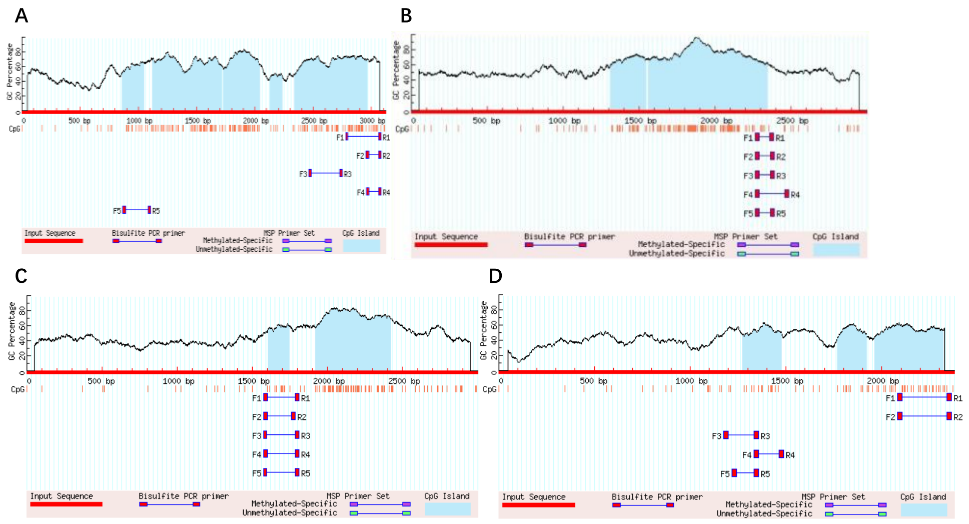


**Supplementary Figure 1. Identification of the CpG islands of four promoter methylation-driven genes (PMDGs)**

(**A**-**D**)The upstream 3kb DNA sequence of the promoter regions of four PMDGs including*DPP6*,*HIST1H4E*, *MTMR7*, and *ZFP28*is extracted from NCBI dataset. The CpG islands (blue segments) are confirmed if CGpercentage is more than 50%.

A

TTGCGCTCGCTTAACCTTGATGCAGCTCGAGGCTTCCCAGTCCAGCT
CAGTTCAGACAGAAAACCTGGCGCGCGCGCGCGCACACACACACGCC
CTCCCCTGGCGTCGCCGCCCGGCCGGGTCCCTGCCCTTAGGGACCA
GAGCGGCGACCGCTGCACCCCGCACCGCCTGCTGGAGGAGCCCCC
GGAGCCGGGGCCGAGCCGCCGGCGTCCCCGAGTGCGCCCCCTGTG
CGTGCCGCCGCGCTGTTGCTCGCAGTGTGCTGGCGCCGAGCTCGGT
GGACACGCGCGCAGTCAGAGCTGCCTCTCGCCCTCGCTAGCTGGGC
TCGCAGCCTCTTCCTCCCTCCCTGGCTCCTGGCTTTTTGTTTAAAGCA
ACACCCACCCTCCATCCAGGCTTTTTTTCTTTCTTTCTTTATTGGTAGC
GGCCAAAAAGAGTTGATTGCTATTGGGATCCGCTGAGTAAAGACACG
GGCAGGGGTGCGCGGAGGTGAGAAAACTGAAGACCTGGAAGATTTTT

B

ATCTCATTTAGTTGTTAAAATATGCCTAAATTTCCTCTTTGGGAACGCA
AGACTTGCAGAGATGACTCCATGGAGAGCGGACTCTGCCGGCGGGA
ACTGGAGTCGTTGGTGACGTCATCCCAGTCTGATCTGTGAAGGGTAG
GGCCAGCAGGCAGCACCAAAGTTCCCGTATGCGCGTTTTCAGTCTTC
ATTTAGGTCCGAATTCCCGGCATATAAGAATACTACCGTCGCTTGTTTT
TCAGATTTTTGCGGCTATTTTCGTTGGTGTGTTGGTCATGTCTGGTCG
CGGCAAAGGCGGAAAGGGACTGGGTAAAGGAGGCGCTAAGCGTCAC
CGTAAGGTCCTGCGAGATAACATCCAGGGCATTACCAAGCC

C

GGGAAAAGTGTCGCCTGCAACAGTGAAGTCGCAGGTGGATGTGAGAT
GCCTGACTTAGGAGCGCGCTTACCAAGGCGCTAACACTGCTGCTATT
ATTTCCACCCGCACCCCGAGCGCTATGCCGGCCGCGCCTGGGATGA
AACACACACATTGAGCCTACAAGACCGTCCTGGGTCTAGAGTGTCTGT
GGCAGCCTTCTTTTCTGCCCCACCCTGAAATCCTAGCTATCCCAGGAC
GCCCTAGAAGGAAGCCCAGGGACGGTGTGGAGCAGCCTGTACTCCC
CATCTCTCTTCCAGGAGGCCACACCCAAAACAAGGCCCTCTTTGTGTC
TCGGAGAACAGTGGCCGTATAGTGCTCCGCCGGCTGCCCCTGTTAAG
AGAGAGCGAGCAGCCCTCGCCCCGGCAACCCCAG

D

TATTTTGGAGGATGGGAGGCCCGTGTAGCCAGAGATAATGGGAGAAG
GTGAGGGGGCACCCAAAACAATCCCACAAGCTCTCTACATCCCCCTT
CCTAGCCCACACACATCACCCATCAGAGTGATCCTCCGGACTCCGGA
GGCCCCCAAGATTGCCTTTCCCTTCCCGGTCGGCAGTGACGCTCCCG
CAAGGAGCTGACGCCCAGGCCCTTAGCACCTGTCACAGCCAAGCACC
CTTTTACTCCGGCCCTGCGGTCCCACCCCTCGCCGCAAGCCCCGCCT
CACCCGGTCTGCCGCTGCGCCTGCGCGACTCCCAGAAGCCTTTGGG
GGGTGGGTGTGTAGTCAGAGCGGCCTCTGCTTCCGGCCACACCCAG
GCCCAGCGTTGGCCA

**Supplementary Figure 2. Selection of CpG islands for bisulfite sequencing polymerase chain reaction**

CpG islands were selected closing to the probe location of HM450K BeadChip.The green segments represent the primerbindingsequence and the yellow segments mean the probe cites.(**A**) Sequence (yellow) of one probe (cg22620221) in the *DPP6* promoter. (**B**) Sequence (yellow) of six probes (cg02656667,cg07140158,cg16282993,cg16706631,cg17123534, and cg19595956) in the *HIST1H4E* promoter. (**C**) Sequence (yellow) of four probes (cg04792712,cg08231493,cg12296772, and cg15600488) in the *MTMR7* promoter. (**D**) Sequence (yellow) of four probes (cg03305181,cg12973930,cg25963041, and cg23850212) in the ZFP28 promoter.

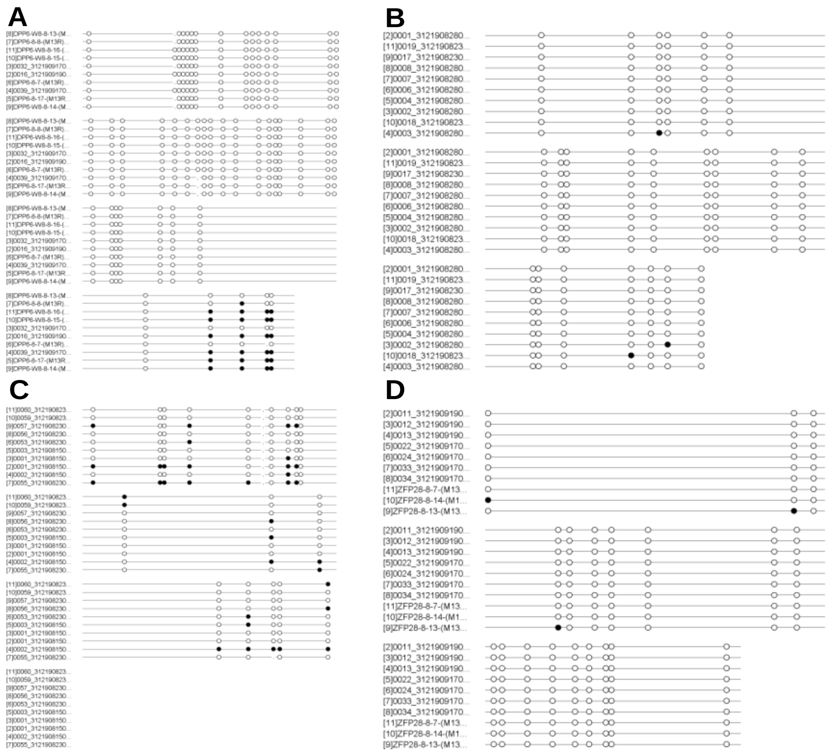

**Supplementary Figure3. Evaluation of the promoter methylation status of four promoter methylation-driven genes**

BiQ Analyzer is used to proceed the original sequencing data and to calculate the numbers of methylated CG sites.(**A**-**D**) The black dots represent the methylated CG sites in the promoters of *DPP6*, *HIST1H4E*, *MTMR7*, and *ZFP28*.

**Supplementary Table 1 Identification of 50 promoter methylation-driven genes**

| Gene | Normal[a] | Tumor[b] | *P*[c] | Fold change[d] | Hazard Ratio[e] | *P* of survival[f] | R value[g] | *P* of correlation[h] |
|---|---|---|---|---|---|---|---|---|
| *FMN2* | 0.069 | 0.371 | <0.001 | 5.37 | -0.26 | 0.013 | -0.43 | <0.001 |
| *PCSK2* | 0.049 | 0.222 | <0.001 | 4.52 | -0.21 | 0.045 | -0.20 | <0.001 |
| *ZFP28* | 0.086 | 0.342 | <0.001 | 3.99 | -0.28 | 0.001 | -0.73 | <0.001 |
| *SRRM4* | 0.088 | 0.325 | <0.001 | 3.70 | 0.60 | 0.015 | -0.29 | <0.001 |
| *HMX2* | 0.098 | 0.338 | <0.001 | 3.44 | 0.58 | 0.011 | -0.21 | <0.001 |
| *RADIL* | 0.069 | 0.237 | <0.001 | 3.43 | -0.26 | 0.012 | -0.43 | <0.001 |
| *NEU1* | 0.057 | 0.189 | <0.001 | 3.30 | -0.16 | 0.046 | -0.65 | <0.001 |
| *ZNF569* | 0.054 | 0.169 | <0.001 | 3.11 | -0.20 | 0.048 | -0.67 | <0.001 |
| *ELMO1* | 0.126 | 0.388 | <0.001 | 3.09 | -0.32 | 0.006 | -0.65 | <0.001 |
| *ZNF879* | 0.060 | 0.185 | <0.001 | 3.07 | -0.33 | 0.016 | -0.68 | <0.001 |
| *FSD1* | 0.085 | 0.261 | <0.001 | 3.05 | -0.21 | 0.049 | -0.25 | <0.001 |
| *RIMS1* | 0.111 | 0.326 | <0.001 | 2.93 | -0.23 | 0.016 | -0.23 | <0.001 |
| *PRKAR2B* | 0.030 | 0.087 | <0.001 | 2.85 | -0.18 | 0.019 | -0.20 | <0.001 |
| *C8orf48* | 0.093 | 0.256 | <0.001 | 2.75 | -0.17 | 0.043 | -0.47 | <0.001 |
| *ZNF71* | 0.085 | 0.234 | <0.001 | 2.74 | -0.29 | 0.012 | -0.70 | <0.001 |
| *ZNF471* | 0.158 | 0.428 | <0.001 | 2.70 | -0.23 | 0.033 | -0.66 | <0.001 |
| *ZNF671* | 0.078 | 0.209 | <0.001 | 2.69 | -0.42 | 0.001 | -0.49 | <0.001 |
| *ZNF781* | 0.149 | 0.395 | <0.001 | 2.65 | -0.24 | 0.030 | -0.55 | <0.001 |
| *ZNF470* | 0.104 | 0.272 | <0.001 | 2.61 | -0.23 | 0.030 | -0.67 | <0.001 |
| *SCAND3* | 0.088 | 0.223 | <0.001 | 2.55 | -0.19 | 0.038 | -0.57 | <0.001 |
| *KIF19* | 0.179 | 0.454 | <0.001 | 2.54 | -0.38 | 0.015 | -0.42 | <0.001 |
| *ALX3* | 0.068 | 0.171 | <0.001 | 2.51 | -0.34 | 0.001 | -0.20 | <0.001 |
| *KCNK12* | 0.162 | 0.406 | <0.001 | 2.51 | -0.23 | 0.043 | -0.31 | <0.001 |
| *SLCO4C1* | 0.107 | 0.265 | <0.001 | 2.48 | -0.18 | 0.038 | -0.49 | <0.001 |
| *KIAA1529* | 0.045 | 0.109 | <0.001 | 2.42 | -0.58 | 0.002 | -0.45 | <0.001 |
| *STXBP5L* | 0.104 | 0.249 | <0.001 | 2.39 | -0.18 | 0.040 | -0.22 | <0.001 |
| *WASF3* | 0.088 | 0.209 | <0.001 | 2.36 | -0.23 | 0.004 | -0.41 | <0.001 |
| *DYNLRB2* | 0.046 | 0.108 | <0.001 | 2.36 | -0.24 | 0.005 | -0.44 | <0.001 |
| *CLDN3* | 0.053 | 0.123 | <0.001 | 2.33 | -0.16 | 0.004 | -0.32 | <0.001 |
| *FBLL1* | 0.116 | 0.265 | <0.001 | 2.29 | -0.32 | 0.015 | -0.28 | <0.001 |
| *RGS22* | 0.179 | 0.410 | <0.001 | 2.29 | -0.20 | 0.024 | -0.40 | <0.001 |
| *C1orf70* | 0.146 | 0.332 | <0.001 | 2.28 | -0.27 | 0.024 | -0.25 | <0.001 |
| *DBC1* | 0.223 | 0.507 | <0.001 | 2.27 | -0.19 | 0.027 | -0.37 | <0.001 |
| *HIST1H4E* | 0.107 | 0.240 | <0.001 | 2.25 | -0.14 | 0.022 | -0.29 | <0.001 |
| *CCDC3* | 0.082 | 0.185 | <0.001 | 2.24 | -0.26 | 0.020 | -0.27 | <0.001 |
| *VASH1* | 0.038 | 0.086 | <0.001 | 2.24 | -0.31 | 0.004 | -0.16 | <0.001 |
| *MPPED1* | 0.101 | 0.225 | <0.001 | 2.23 | 0.60 | 0.017 | -0.19 | <0.001 |
| *ADCY5* | 0.147 | 0.325 | <0.001 | 2.22 | -0.15 | 0.013 | -0.31 | <0.001 |
| *MTMR7* | 0.247 | 0.545 | <0.001 | 2.21 | -0.31 | 0.014 | -0.50 | <0.001 |
| *RLTPR* | 0.042 | 0.092 | <0.001 | 2.17 | -0.39 | 0.007 | -0.18 | <0.001 |
| *ALK* | 0.056 | 0.122 | <0.001 | 2.17 | -0.20 | 0.019 | -0.29 | <0.001 |
| *LOC642597* | 0.171 | 0.370 | <0.001 | 2.16 | -0.24 | 0.034 | -0.24 | <0.001 |
| *HOXB4* | 0.154 | 0.333 | <0.001 | 2.16 | -0.15 | 0.050 | -0.41 | <0.001 |
| *ZNF625* | 0.078 | 0.167 | <0.001 | 2.15 | -0.32 | 0.045 | -0.56 | <0.001 |
| *TRPC6* | 0.187 | 0.395 | <0.001 | 2.11 | -0.07 | 0.034 | -0.53 | <0.001 |

| | | | | | | | | |
|---|---|---|---|---|---|---|---|---|
| *LINGO3* | 0.083 | 0.175 | <0.001 | 2.10 | 0.56 | 0.006 | -0.18 | <0.001 |
| *GRM6* | 0.258 | 0.536 | <0.001 | 2.08 | -0.23 | 0.025 | -0.35 | <0.001 |
| *DPP6* | 0.244 | 0.502 | <0.001 | 2.06 | -0.20 | 0.028 | -0.55 | <0.001 |
| *EFHA2* | 0.064 | 0.130 | <0.001 | 2.04 | -0.22 | 0.040 | -0.57 | <0.001 |
| *RTP4* | 0.306 | 0.109 | <0.001 | 0.36 | 0.26 | 0.003 | -0.44 | <0.001 |

[a]Theaverage β-value of the genetic promoter in normal tissues (GSE49149).

[b]The average β-value of the genetic promoter in tumor tissues (GSE49149).

[c]*P* value of t-test of β-valuebetween normal and tumor tissues (GSE49149).

[d]Fold change of β-value (tumor versus normal tissues, GSE49149).

[e]Hazard ratio of univariate Cox regression analyses according to mRNA expression (high- versus low-level groups based on median value, PDAC cohort of TCGA).

[f]*P* value of univariate Cox regression analyses (PDAC cohort of TCGA).

[g]Correlation coefficientbetweenβ-value and mRNA expression (PDAC cohort of TCGA).

[h]*P* value of the correlation between β-value and mRNA expression (PDAC cohort of TCGA).
